# GnRH-(1–5) Inhibits TGF-β Signaling to Regulate the Migration of Immortalized Gonadotropin-Releasing Hormone Neurons

**DOI:** 10.3389/fendo.2018.00045

**Published:** 2018-02-20

**Authors:** Darwin O. Larco, Bradly M. Bauman, Madelaine Cho-Clark, Shaila K. Mani, T. John Wu

**Affiliations:** ^1^Department of Obstetrics and Gynecology, Uniformed Services University of the Health Sciences, Bethesda, MD, United States; ^2^Department of Molecular, Baylor College of Medicine, Houston, TX, United States; ^3^Department of Cellular Biology and Neuroscience, Baylor College of Medicine, Houston, TX, United States

**Keywords:** G protein-coupled receptor 173, gonadotropin-releasing hormone, G protein-coupled receptors, migration, EP24.15

## Abstract

Gonadotropin-releasing hormone (GnRH) neurons originate outside the central nervous system (CNS) in the nasal placode where their migration to the basal forebrain is dependent on the integration of multiple signaling cues during development. The proper migration and establishment of the GnRH neuronal population within the CNS are critical for normal pubertal onset and reproductive function. The endopeptidase EP24.15 is expressed along the migratory path of GnRH neurons and cleaves the full-length GnRH to generate the metabolite GnRH-(1–5). Using the GN11 cell model, which is considered a pre-migratory GnRH neuronal cell line, we demonstrated that GnRH-(1–5) inhibits cellular migration in a wound closure assay by binding the orphan G protein-coupled receptor 173 (GPR173). In our current experiments, we sought to utilize an *in vitro* migration assay that better reflects the external environment that migrating GnRH neurons are exposed to during development. Therefore, we used a transwell assay where the inserts were coated with or without a matrigel, a gelatinous mixture containing extracellular matrix (ECM) proteins, to mimic the extracellular environment. Interestingly, GnRH-(1–5) inhibited the ability of GN11 cells to migrate only through ECM mimetic and was dependent on GPR173. Furthermore, we found that GN11 cells secrete TGF-β1, 2, and 3 but only TGF-β1 release and signaling were inhibited by GnRH-(1–5). To identify potential mechanisms involved in the proteolytic activation of TGF-β, we measured a panel of genes implicated in ECM remodeling. We found that GnRH-(1–5) consistently increased tissue inhibitors of metalloproteinase 1 expression, which is an inhibitor of proteinase activity, leading to a decrease in bioactive TGF-β and subsequent signaling. These results suggest that GnRH-(1–5) activating GPR173 may modulate the response of migrating GnRH neurons to external cues present in the ECM environment *via* an autocrine-dependent mechanism involving TGF-β.

## Introduction

The decapeptide gonadotropin-releasing hormone (GnRH) is a central regulator of reproduction mediated by the well-characterized GnRHR (1, 2). In the extracellular space, GnRH is enzymatically processed by the zinc metalloendopeptidase EC3.4.24.15 (EP24.15) to generate the metabolite, GnRH-(1–5) (3, 4). Interestingly, we have previously demonstrated that the metabolic product of GnRH, GnRH-(1–5), is biologically active through identifying its ability to regulate GnRH-related functions. These include regulating GnRH mRNA expression (5) and reproductive behavior in female rats (6). Furthermore, our studies using immortalized GnRH neurons, the GN11 cell, also suggest that GnRH-(1–5) may also serve a developmental function by regulating the migration of GnRH neurons *via* the activation of the orphan receptor, G protein-coupled receptor 173 (GPR173) (7).

During development, GnRH neurons are born outside the central nervous system (CNS) and migrate along the vomeronasal nerve to subsequently target the basal forebrain by traversing the cribriform plate (8). Within this migratory path, EP24.15 is expressed and can mediate the conversion of GnRH to GnRH-(1–5) to potentially regulate the migration rate of GnRH neurons (9). Our previous studies using GN11 cells suggest that GnRH-(1–5) activating GPR173 may contribute to the maturation or aid in the transition of GnRH neurons from the olfactory region to the CNS. Furthermore, during this transition, migrating GnRH neurons need to appropriately adapt to the signaling cues present in the changing extracellular environment (10). In GN11 cells, GnRH-(1–5) inhibited cellular migration, which during development may serve to modulate the communication between migrating GnRH neurons and the extracellular environment (7, 11).

G protein-coupled receptor 173 is a member of the subfamily of G protein-coupled receptors (GPCRs) called the Super Conserved Receptor Expressed in Brain (SREB) family, which also includes the orphan receptors GPR27 and GPR85 (12). The SREB proteins are considered to bind small aminergic peptide ligands and are expressed primarily in the brain and genital organs (12). Our studies indicate that GPR173 may play a role in mediating the migration of GnRH neurons during development. There is already a growing list of GPCRs implicated in the proper migration of GnRH neurons including PROKR (13) and CXCR4 (14, 15). With regard to GPR173, migrating GnRH neurons progressively produce increasing levels of GnRH (16, 17), which would subsequently be processed by EP24.15 to generate GnRH-(1–5) and bind GPR173. The interaction of GnRH-(1–5) and GPR173 may serve to modulate the migratory rate of GnRH neurons as they target the basal forebrain. However, further investigation is warranted into the mechanism of GnRH-(1–5) and GPR173 regulating GnRH neuronal migration.

In this study, we investigated the mechanism of GnRH-(1–5) to regulate the migratory response of GN11 cells in the presence of an extracellular matrix (ECM) environment. We used a transwell assay coated with a matrigel, a gelatinous mixture containing ECM proteins to mimic the extracellular environment. Furthermore, we examined whether GnRH-(1–5) regulates chemokine and growth factor release to implicate their role in the effects of GnRH-(1–5) on migration. We found that GnRH-(1–5) inhibits TGF-β activation and signaling to inhibit the migration of GN11 cells.

## Materials and Methods

### Reagents and Cell Culture

GnRH-(1–5) was purchased from Bachem (Torrance, CA, USA) and reconstituted to 10 mM in distilled water and stored in 12 µL aliquots at −80°C. GN11 cells (18) generously donated by Dr. Sally Radovick (Robert Wood Johnson Medical School, Rutgers University, New Brunswick, NJ, USA) were grown in Dulbecco’s modified Eagle’s medium (DMEM; Mediatech Inc., Herndon, VA, USA) without antibiotics and supplemented with 7% fetal bovine serum (FBS; Hyclone, Logan, UT, USA), 3% newborn calf serum (Hyclone), 25 mM glucose, and 5 mM l-glutamine (7, 11, 19). Cells were maintained at 37°C in an atmosphere with 5% CO_2_. In addition, all experiments were conducted at 37°C in an atmosphere with 5% CO_2_. All GN11 cells used for experimentation were below passage 25. GN11 cells originated from an olfactory tumor extracted from a male mouse (18). The antibodies used in this study were specific for the TGF-βRI (1:1,000; Cat. No. SC-9048, Santa Cruz Biotechnology, Dallas, TX, USA), TGF-βRII (1:1,000; Cat. No. SC-400, Santa Cruz Biotechnology), GAPDH (1:2,000; Cat. No. SC-25778, Santa Cruz Biotechnology), and anti-rabbit IgG conjugated to horseradish peroxidase (1:10,000; Cat. No. 170-6515, Bio-Rad, Hercules, CA, USA).

### Embryonic Nasal Tissue

RNA extracted from a mouse nasal region was obtained at embryonic day (ED) 12.5. This sample was generously donated by Dr. Susan Wray (National Institutes of Health, Bethesda, MD, USA) and described previously by us for detection of GPR173 (7) and by Wray and colleagues (20). All experimental procedures were conducted in accordance with the National Institutes of Health guidelines (Guide for the Care and Use of Laboratory Animals, NIH Publication No. 85-23, revised 1996) and were approved by the Uniformed Services University of the Health Sciences Institutional Animal Care and Use Committee.

### Chemokine and TGF-β Bioplex Assay

GN11 cells were plated on six-well plates and allowed to grow until 80% confluency. Subsequently, the media were changed to serum-free media and starved for at least 24 h prior to treatments. Cells were treated with or without 100 nM GnRH-(1–5) in 10% charcoal-stripped FBS (volume of 700 μL/well) for 5 min or 24 h. At the end of each experiment, the cells were placed on ice and the conditioned media collected and immediately frozen at −80°C for future analysis of both chemokine and TGF-β content. Cell lysates were also collected to determine protein concentration using a Pierce protein BCA assay (Thermo Fisher; Waltham, MA, USA). The chemokines CXCL1, CXCL2, CXCL9, CXCL10, CCL3, CCL4, CCL5, and MCSF were measured by the multiplex assay Milliplex Mouse Chemokine Panel (Cat. # MPXMCYTO-70K; EMD Millipore, Billerica, MA, USA) according to manufacturer’s instructions. The levels of TGF-β1, 2, and 3 were measured by the multiplex assay Bio-Rad Pro TGF-β Assay 3-plex (Cat. # 171W4001M; Hercules, CA, USA) according to the provided protocol. All multiplexing assays were performed using the Bio-Plex 200 Luminex assay system (Bio-Rad; Hercules, CA, USA).

### TGF-β-Activated Luciferase Assay

The conditioned media of GN11 cells treated with ±100 nM GnRH-(1–5) for 5 min or 24 h were used to measure bioactive TGF-β using a mink lung epithelial cell (MLEC) line stably expressing the TGF-β-responsive plasminogen activator-1 luciferase reporter (21, 22). Briefly, MLECs were plated on 24-well plates and allowed to grow until 80% confluent in DMEM supplemented with 10% FBS and 80 µg/mL G418 (Sigma; St. Louis, MO, USA). Subsequently, the media were changed to serum-free DMEM supplemented with 0.1% insulin/transferrin/sodium selenite (Cat. # 354352; Corning, Corning, NY, USA), and the MLECs were allowed to incubate overnight. The following day MLECs were treated with 20 µL of conditioned media harvested from treated GN11 cells. After 16 h, MLECs were lysed with Promega Passive Lysis Buffer (Madison, WI, USA) followed by the addition of the luciferase substrate. Samples were subsequently analyzed using a Synergy H1 Hybrid Plate Reader (BioTek; Winooski, VT, USA), and values were expressed as a percentage change relative to VEH-treated GN11 cells. Each treatment condition was assayed in three replicates, and the experiments were performed at least five times.

### Focused Array Pathway Analysis

Analysis of genes involved in ECM remodeling in GN11 cells was performed using a pre-designed PrimePCR Pathway Plate (Cat. No. 10029332; Bio-Rad). Total RNA was extracted using TRIzol reagent (Invitrogen) 2 h post treatment with GnRH-(1–5) and purified using RNeasy Mini Kit (Qiagen) according to manufacturer’s instructions. A total of 2 µg of RNA were reverse transcribed using the iScript Advanced cDNA Synthesis kit (Bio-Rad). SsoAdvanced Universal SYBR Green Supermix (Bio-Rad) and cDNA were added to each well up to a total 20 µL according to manufacturer’s instructions. All samples were assayed using the CFX Connect Real-time System (Bio-Rad). The cycling conditions were performed as follows: initial denaturation and enzyme activation at 95°C for 2 min followed by 40 cycles of denaturation (95°C, 5 s), annealing, and reading (60°C, 30 s). Melt curve analysis was performed to ensure the presence of a single amplicon.

### Droplet Digital PCR (ddPCR)

Droplet digital PCR relies on water-emulsion technology to form ~20,000 nL-sized droplets for absolute quantitation of gene expression. The generation of droplets for our experiments was performed using the Automated Droplet Generator (AutoDG; Bio-Rad). Each sample was prepared using 2x QX200 ddPCR EvaGreen Supermix (Bio-Rad), forward and reverse primers (Table 1; final concentration of 200 nM), template (variable volume; 200 pg cDNA), and ultra-pure H_2_O for a final volume of 22 µL. Samples were loaded onto a 96-well PCR plate (Eppendorf, Hamburg, Germany), which was then loaded into the AutoDG instrument for droplet generation. Generated droplets were transferred by the AutoDG to a new 96-well PCR plate which was then heat-sealed with a pierceable foil using a PX1 PCR Plate Sealer (Bio-Rad). The plate was loaded into a conventional thermal cycler (C1000 Touch, Bio-Rad), and the following thermal cycling conditions were used: initial denaturation and enzyme activation at 95°C for 5 min, 40 cycles of denaturation (95°C, 30 s) and annealing/extension (55°C, 1 min), and final signal stabilization steps at 4°C (5 min) and then 90°C (5 min). After thermal cycling, the 96-well plate was loaded into the QX200 Droplet Reader (Bio-Rad), and the droplets were automatically aspirated and read. All data were analyzed using the provided analysis software (QuantaSoft, Bio-Rad).

**Table 1:** Primer sequences used for quantitative RT-PCR and droplet digital PCR.

Gene	Accession number	Primer sequence	Amplicon size (bp)
GPR173	NM_027543.3	(F) 5′-CAGCTAGTGGGAGGAAGCTGCT-3′	187
		(R) 5′-TGCTGAGCTACACCTGCAAATGGG-3′	
Cyclophilin A	NM_008907.1	(F) 5′-CCAAACACAAACGGTTCCCA-3′	94
		(R) 5′-TGCCTTCTTTCACCTTCCCAA-3′	
TIMP1	NM_001044384.1	(F) 5′-CACACCAGAGCAGATACCATGA-3′	200
		(R) 5′-CGCTGGTATAAGGTGGTCTCG-3′	
TIMP2	NM_011594.3	(F) 5′-TGCAGACGTAGTGATCAGAGC-3′	163
		(R) 5′-AGAGGGGGCCGTGTAGATAA-3′	
TIMP3	NM_011595.2	(F) 5′-CAACTCCGACATCGTGATCC-3′	130
		(R) 5′-CACGTGGGGCATCTTACTGA-3′	
TBP	NM_013684.3	(F) 5′-CCTATCACTCCTGCCACACC-3′	161
		(R) 5′-ATGACTGCAGCAAATCGCTTG-3′	

### Western Blot Analysis

Total protein lysates were harvested from GN11 cells with O’dell’s lysis buffer [10 mM EGTA, 10 mM EDTA, 80 µM Na_2_MoO_4_, 5 mM NaPO_4_, 1 mM Na_3_VO_4_, 1 mM phenylmethylsulfonyl fluoride, 4 mM *p*-nitrophenyl phosphate, 1% Triton X-100, and Sigma Inhibitor Cocktail I, II, and Roche Complete Protease Inhibitor (Roche; Indianapolis, IN, USA)]. Cell lysates were briefly sonicated, and the supernatant (30 µg) was subjected to SDS-PAGE (4–12%, Lonza) after centrifugation (20,000 × *g* for 20 min at 4°C). After electrophoresis, the gel was transferred onto a polyvinyl difluoride membrane using a Trans-Blot Turbo Transfer System (BioRad) and incubated in 5% non-fat dry milk at room temperature for 1 h to block non-specific antibody binding. The membrane was then rinsed with TBS-Tween (TBST) and incubated with the primary antibody overnight at 4°C. The membrane was subsequently rinsed 3 × 10 min with TBST, incubated with the secondary antibody at an appropriate dilution conjugated to horseradish peroxidase (Bio-Rad) for 1 h at room temperature, then rinsed 5 × 15 min with TBST. The blots were visualized by chemiluminescence (Millipore, Billerica, MA, USA) using a Fujifilm LAS-3000 imager (Fujifilm; Stamford, CT, USA).

### Gelatin Zymography

The effect of GnRH-(1–5) on matrix metalloproteinase (MMP)-2 and 9 activity in conditioned medium was determined by zymography (23–27). Serum-starved GN11 cells in six-well plates (at 80% confluency) were treated with 700 µL of ±100 nM GnRH-(1–5) in 10% charcoal-stripped FBS. After 1 h, 25 µL of the conditioned media was diluted 1:1 with sample buffer (Bio-Rad) and subjected to electrophoresis in 10% gelatin polyacrylamide gels (Bio-Rad) under non-reducing conditions. Proteins within the gel were renatured by removing the SDS *via* washing for 30 min in 2.5% Triton X-100. Gels were developed in collagenase buffer [50 mM Tris-HCl (pH 7.5), 200 mM NaCl, 5 mM CaCl_2_, and 0.02% Brij-35] for 72 h and stained with 0.5% Coomassie blue R-250 (Bio-Rad). After destaining gels in 30% methanol and 10% acetic acid, areas of digestion were digitized using the Fujifilm LAS-3000 imager (Fujifilm; Stamford, CT, USA). MMP activity is represented by clear bands and quantified with Fujifilm Image Gauge Software (Valhalla, NY, USA).

### Matrigel Invasion Assay

The invasion assay was conducted using transwell inserts with 8 µm pores coated with a reduced growth factor matrigel (BD Biosciences; Palo Alto, CA, USA). The matrigel is a proteinaceous mixture composed of collagen IV, laminin, entactin, and proteoglycan to resemble the constituents of the ECM. Cells exhibiting a more invasive and migratory phenotype will be better able to digest the matrigel to traverse insert. In our model, GN11 cells were grown in six-well plates until 80% confluent and allowed to serum-starve for at least 24 h. Subsequently, the cells were harvested with dissociation buffer (2 mM EDTA diluted in 0.1 M phosphate-buffered saline). The cells were briefly centrifuged (5 min at 500 g) to remove the dissociation buffer and resuspended in 2 mL of DMEM per well of a six-well plate. A total of 250 µL of cell suspension was added to the upper chamber of the transwell assay followed by 250 µL of DMEM with or without 200 nM GnRH-(1–5) (final concentration of 100 nM). The lower chamber contained 750 µL of 10% charcoal-stripped FBS as a chemoattractant. Chambers were subsequently maintained at 37°C in an atmosphere with 5% CO_2_. After 24 h, cells that had not migrated through the matrigel-coated inserts were gently removed using cotton swabs, and the invading cells on the underside of the membrane were fixed and stained with 0.5% crystal violet in 10% buffered paraformaldehyde. Stained GN11 cells were imaged at 20× with the Leica AF6000 microscope and counted using Image J (NIH, Bethesda, MD, USA). Multiple images were taken of each insert and averaged. Values were expressed as a percentage change relative to VEH (24, 28, 29). Each condition was assayed in duplicate and represents at least three independent experiments.

### GPR173 Silencing by siRNA

GN11 cells were plated at 20% confluency and transfected with a cocktail of siRNA (25 nM) specific to GPR173 (SMARTpool; Cat. No. L-059639-00-0005, Dharmacon, Lafayette, CO, USA). Control cells were transfected with a non-targeting siRNA mixture (25 nM) (SMARTpool; Cat. No. D-001810-10-05, Dharmacon). Dharmafect4 (Dharmacon) was used as the transfection reagent. All treatments were done in 10% charcoal-stripped dextran-treated FBS (Atlanta Biologicals; Lawrenceville, GA, USA). Cells were incubated with the siRNA treatments for 48 h and then serum-starved for at least 24 h prior to experimentation.

### Quantitative RT-PCR (qPCR)

The efficiency of GPR173 silencing by siRNA was measured in GN11 cells by qPCR. Total RNA was harvested 48 h post-transfection with siRNA as described above. A total of 2 µg of RNA was reverse transcribed using the Maxima First Strand cDNA synthesis kit (Fermentas; Glen Burnie, MD, USA), and the cDNA was analyzed by qPCR using the FAST SYBR Green Mastermix (Cat. No. 330609, Qiagen, Valencia, CA, USA) using 200 nM of the appropriate primer pair. All samples were assayed in duplicate using the CFX Connect Real-time System (Bio-Rad; Hercules, CA, USA). The qPCR conditions were performed as follows: initial denaturation and enzyme activation at 95°C for 10 min followed by 40 cycles of denaturation (95°C, 15 s), annealing, and reading (60°C, 30 s). Melt curve analyses was conducted after each qPCR reaction to demonstrate the presence of a single amplicon. Additionally, qPCR products were visualized by agarose gel electrophoresis (1%) using the GelStar Nucleic Acid Gel Stain (Lonza; Rockland, ME, USA) under UV light and verified by sequencing. Primers specific to GPR173 and the reference gene Cyclophilin A are shown in Table 1. qPCR conditions were optimized to produce a greater than 95% efficiency for both primer pairs as determined by a 10-fold serial dilution of cDNA (29). The delta delta Ct method was used to measure fold change in GPR173 expression relative to levels obtained from VEH-treated cells. This experiment was repeated over three consecutive passages.

### Statistical Analysis

Statistics were conducted using the analysis software GraphPad Prism 6 (GraphPad Software, Inc.; La Jolla, CA, USA). For most experiments, normalized data were analyzed using a paired *t*-test to determine significance. Studies using siRNA, data were analyzed by a one-way ANOVA followed by Fisher’s least significant difference *post hoc* test. ddPCR data were presented as target copy number/TATA box binding protein copy number and analyzed by a non-parametric paired-sample Wilcoxon test. A value of *p* < 0.05 was considered significant.

## Results

### Effect of GnRH-(1–5) on GN11 Cellular Invasion

**Figure 1:**
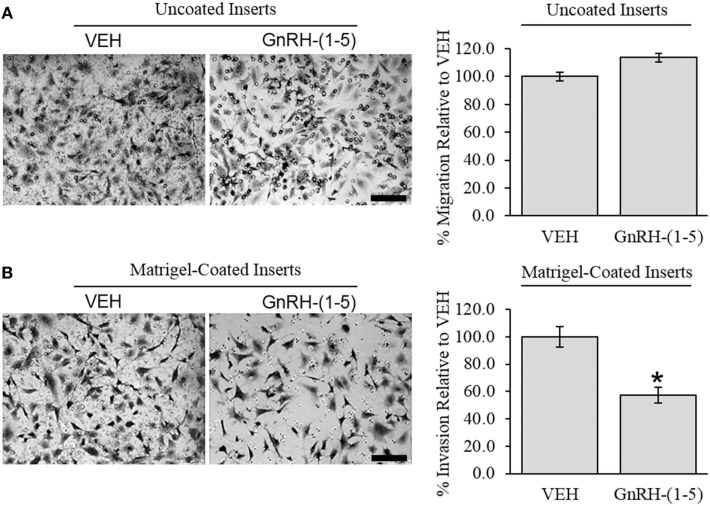
Effect of GnRH-(1–5) on GN11 cellular invasion. The migration of GN11 cells was measured in response to GnRH-(1–5) using a transwell assay with inserts either coated with matrigel or not. **(A)** The left panel indicates representative photomicrographs of migrated GN11 cells treated with VEH or 100 nM GnRH-(1–5) for 24 h through uncoated inserts. The right panel is a quantification of the results and demonstrated that GnRH-(1–5) treatment did not have an effect on the migration of GN11 cells exposed to empty inserts. **(B)** The lower panels indicate that cells exposed to a matrigel environment and treated with 100 nM GnRH-(1–5) for 24 h had decreased invasion relative to respective VEH-treated cells. The results are averages from four independent experiments. Data were normalized to VEH-treated cells and expressed as a percentage change. A paired *t*-test was conducted to determine significance. Scale bar indicates 100 µm. *p < 0.05 relative to VEH.

**Figure 2:**
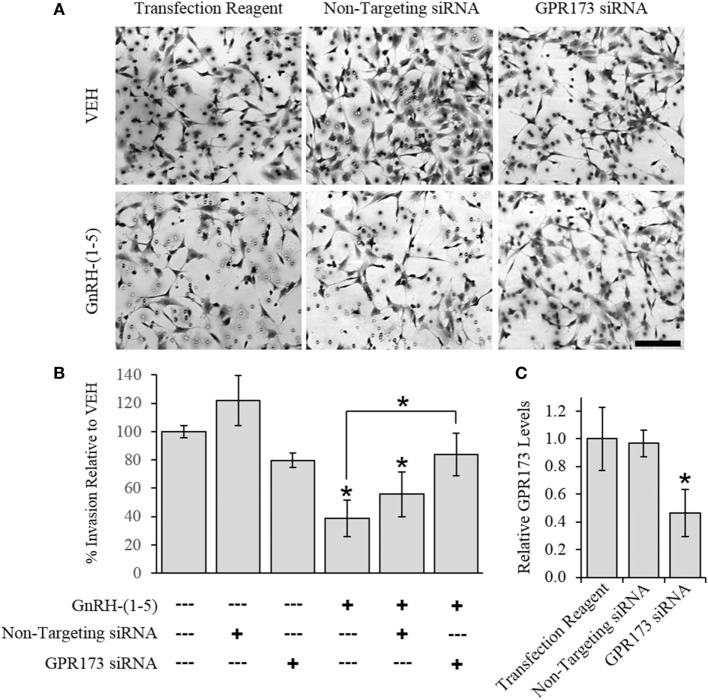
Effect of G protein-coupled receptor 173 (GPR173) silencing on the GnRH-(1–5)-mediated inhibition of invasion. GPR173 was downregulated by siRNA in GN11 cells, and the effect of GnRH-(1–5) to regulate invasion was investigated. **(A)** Upper panel shows representative photomicrographs of migrated GN11 cells treated with the indicated siRNA conditions with and without 100 nM GnRH-(1–5). **(B)** Lower panel indicates the quantification of the invasion assay results. The effect of GnRH-(1–5) to inhibit invasion was blocked in cells exposed only to siRNA targeting GPR173 levels. **(C)** GN11 cells exposed to GPR173 siRNA had a greater than 60% reduction in GPR173 mRNA levels (*n* = 3). Migration assay data are representative of three independent experiments and were analyzed by a one-way ANOVA followed by a least significant difference *post hoc* test. Scale bar indicates 100 µm. *p < 0.05 relative to VEH.

To determine the effect of GnRH-(1–5) on GN11 cellular invasion, a transwell migratory assay was implemented where the inserts were coated with and without a matrigel. The matrigel is a proteinaceous mixture of ECM proteins that mimics the extracellular environment. GN11 cells treated with 100 nM GnRH-(1–5) had no effect in empty inserts but significantly inhibited the ability of GN11 cells to traverse the matrigel environment (Figures 1A,B). Subsequently, we determined whether the effect of GnRH-(1–5) on invasion was dependent on GPR173 using a siRNA approach. The expression of GPR173 was decreased by GPR173-specific siRNA, and the effect of GnRH-(1–5) on GN11 cellular invasion was assessed. GnRH-(1–5) treatment significantly inhibited the cellular invasion of GN11 cells, which was reversed in cells pre-treated with GPR173-specific siRNA (Figures 2A,B). GPR173 downregulation was confirmed by qPCR demonstrating greater than 60% knockdown (Figure 2C).

### Effect of GnRH-(1–5) on Chemokine Release

**Figure 3:**
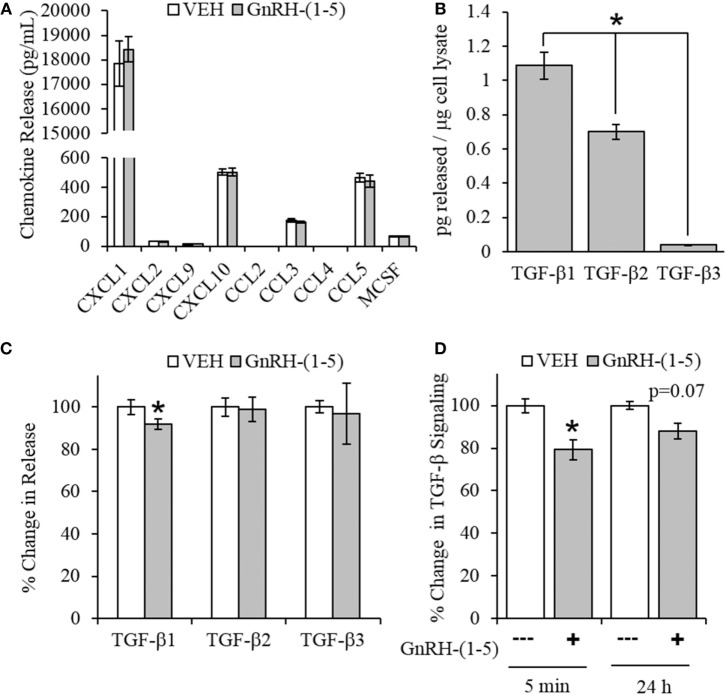
Effect of GnRH-(1–5) on chemokine release and TGF-β signaling. **(A)** GN11 cells were treated with 100 nM GnRH-(1–5) for 24 h, and the media were collected to measure chemokine release. GnRH-(1–5) had no effect on the release of CXCL1, CXCL2, CXCL9, CXCL10, CCL3, CCL4, CCL5, and MCSF (*N* = 3). **(B)** We also measured TGF-β1, 2, and 3 and found that GN11 cells significantly secrete more TGF-β1 than the other isoforms (*N* = 3). **(C)** Next, we determined whether GnRH-(1–5) regulates TGF-β release. GN11 cells were treated with 100 nM GnRH-(1–5) for 24 h, and the media were measured for total TGF-β1, TGF-β2, and TGF-β3 release. GnRH-(1–5) treatment inhibited TGF-β1 release but not TGF-β2 and TGF-β3 (*N* = 4). **(D)** Similarly, using an established TGF-β bioassay to measure bioactive TGF-β, conditioned media collected from GN11 cells treated with GnRH-(1–5) for 5 min and 24 h had decreased TGF-β activity (*N* = 5–6). A paired *t*-test was performed to determine significance. *p < 0.05 relative to VEH.

Chemokines are secreted factors that are implicated in the regulation of cellular migration and invasion, including the migratory response of GN11 cells (30). Therefore, we measured chemokine release using a multiplex approach to quantify multiple chemokines from the same sample. GN11 cells were treated with 100 nM GnRH-(1–5) for 24 h, and the conditioned media were collected for analysis. GnRH-(1–5) treatment had no significant effect on the release of CXCL1, CXCL2, CXCL9, CXCL10, CCL3, CCL4, CCL5, and MCSF (Figure 3A).

### Effect of GnRH-(1–5) on TGF-β Release

**Figure 4:**
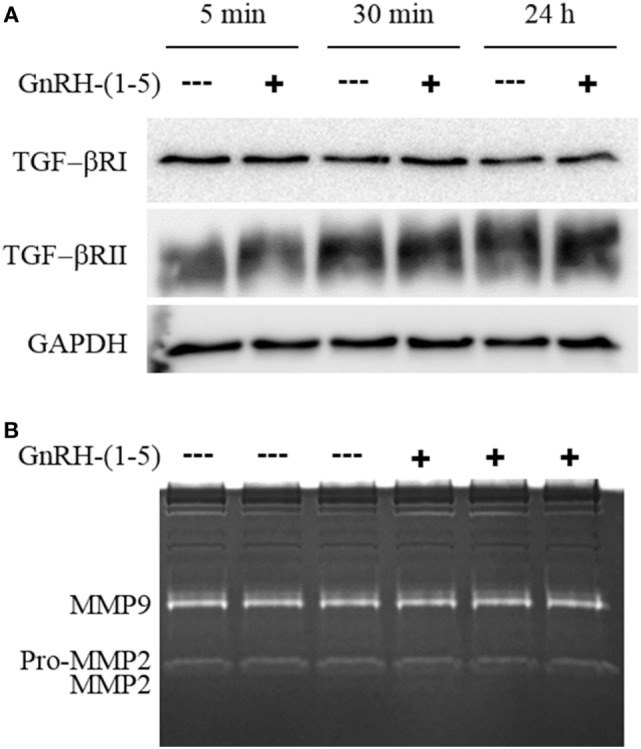
Effect of GnRH-(1–5) on TGF-βR levels and matrix metalloproteinase (MMP) activity. **(A)** Time course studies were conducted to confirm TGF-βRI and TGF-βRII expression and whether GnRH-(1–5) treatment regulates receptor levels. Western blot analysis revealed the expression of both TGF-βRI and TGF-βRII in GN11 cells, but neither was regulated by GnRH-(1–5) treatments. **(B)** Subsequently, we measured MMP-2 and MMP-9 activity by gel zymography to determine whether the decrease observed in bioactive TGF-β is mediated by these proteinases. Conditioned media isolated from GN11 cells treated with or without 100 nM GnRH-(1–5) did not change the activity of MMP-2 and MMP-9. Data are representative of three independent experiments and were analyzed by a paired *t*-test.

Since there were no differences in chemokine release in response to GnRH-(1–5), we measured the levels of TGF-β release. TGF-β signaling has been implicated in the development of the CNS including the regulation of neuronal migration (31). To measure all TGF-β isoforms, we used a multiplex approach that can quantitate the levels of TGF-β1, 2, and 3. GN11 cells significantly produce more TGF-β1 than TGF-β2 and TGF-β3 (Figure 3B). Furthermore, GN11 cells treated with 100 nM GnRH-(1–5) for 24 h had significantly less total TGF-β1 compared to the other two isoforms (Figure 3C). To determine whether the modest but significant decrease in TGF-β1 release was biologically effective in decreasing TGF-β signaling, we used the MLEC line that stably expresses the TGF-β-responsive plasminogen activator-1 luciferase reporter and has been previously described to measure bioactive TGF-β1 in the pM range (21, 22). MLECs were treated with conditioned media of GN11 cells that were treated with or without 100 nM GnRH-(1–5) for 5 min or 24 h. GnRH-(1–5) treatment significantly decreased activation of the reporter construct at 5 min, indicating that the levels of bioactive TGF-β were diminished (Figure 3D). In addition, there was a decrease at the longer time point of 24 h; however, this effect was not significant (Figure 3D). Subsequently, we sought to determine whether GN11 cells express the TGF-β receptors and whether there were changes in total receptor expression, which may contribute to the decrease in TGF-β signaling. We conducted time course studies (5 min, 30 min, and 24 h) in GN11 cells treated with and without 100 nM GnRH-(1–5) and measured TGF-βRI and TGF-βRII by Western blot. In addition, time course studies were conducted to determine changes in TGF-βRI and TGF-βRII levels in response to GnRH-(1–5) treatment. GN11 cells express both TGF-βRI and TGF-βRII, but neither receptor is significantly regulated by GnRH-(1–5) treatment (Figure 4A).

### Effect of GnRH-(1–5) on MMP-2/9 Activity

Matrix metalloproteinases, especially MMP-2, have been implicated in the proteolytic activation of TGF-β (32, 33). Therefore, we sought to determine whether GnRH-(1–5) inhibits MMP-2 or MMP-9 activity to decrease the availability of bioactive TGF-β. We measured MMP-2 and MMP-9 release and activity by gel zymography from conditioned media of GN11 cells treated with or without 100 nM GnRH-(1–5) for 1 h. No significant changes were observed with GnRH-(1–5) treatment (Figure 4B).

### Effect of GnRH-(1–5) on ECM Remodelers

**Figure 5:**
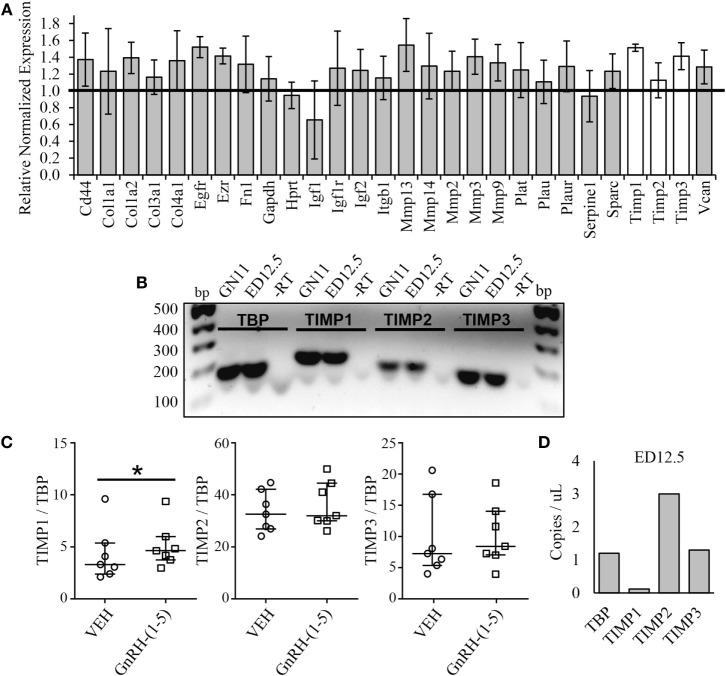
Effect of GnRH-(1–5) on extracellular matrix (ECM) remodelers. **(A)** A focused array was implemented to determine changes in ECM remodelers in response to GnRH-(1–5). GN11 cells were treated with 100 nM GnRH-(1–5) for 2 h and the RNA isolated for these studies. GnRH-(1–5) treatment modestly increased the levels of tissue inhibitors of tissue inhibitors of metalloproteinase (TIMP) 1 and TIMP3 but not TIMP2 (*N* = 2). **(B)** TIMP1, 2, and 3 expression was confirmed by gel electrophoresis in GN11 cells and nasal tissue extracted from a mouse at embryonic day (ED) 12.5. A reaction in which the reverse transcriptase is absent was used as a negative control (−RT). **(C)** To confirm the PCR array results, droplet digital PCR (ddPCR) was performed to measure copy numbers of TIMP1, 2, and 3 in GN11 cells in response to GnRH-(1–5) treatment. GnRH-(1–5) treatment significantly increased TIMP1 but not TIMP2 or TIMP3 levels (*N* = 6). A non-parametric paired-sample Wilcoxon test was used to determine significance. The medians and interquartile ranges (error bars) are indicated for each group. **(D)** Likewise, ddPCR was performed on nasal tissue from a mouse (ED12.5) to quantitate the relative abundance of each TIMP in this region (*N* = 1).

Since we did not see changes in MMP-2 or MMP-9 activity, it is likely that GnRH-(1–5) may regulate ECM remodelers involved in the activation of TGF-β. Thus, we implemented a high-throughput focused array assay that measures the expression of 43 genes implicated in ECM remodeling. GN11 cells were treated with or without 100 nM GnRH-(1–5) for 2 h, and subsequently the RNA was isolated and analyzed using the focused array. GN11 cells did not express any of the kallikrein family members, which are a group of serine proteases (data not shown). GnRH-(1–5) treatment modestly increased the levels of tissue inhibitors of metalloproteinase (TIMP) 1 and TIMP3 but not TIMP2 (Figure 5A). TIMPs are inhibitors of proteinase activity, which may play a role in regulating proteinases involved in the proteolytic cleavage of latent TGF-β to active TGF-β. Therefore, we confirmed TIMP1, 2, and 3 expressions in GN11 cells and nasal tissue extracted from a mouse at ED 12.5 (Figure 5B). Furthermore, statistical analysis was performed on ddPCR measuring copy numbers of TIMP1, 2, and 3 in GN11 cells in response to GnRH-(1–5) treatment to confirm the PCR array data. GnRH-(1–5) treatment significantly increased TIMP1 levels but not TIMP2 or TIMP3 (Figure 5C). Likewise, ddPCR was performed on nasal tissue from a mouse (ED12.5) to quantitate the relative abundance of each TIMP in this region (Figure 5D).

## Discussion

Our previous work demonstrated that GnRH-(1–5) inhibits the migration of GN11 cellular migration (7, 11). In our initial studies, we implemented a wound closure assay to assess the effect of GnRH-(1–5) on GN11 cells and found that the effects of GnRH-(1–5) are dependent on GPR173. We also demonstrated that the effects of GnRH-(1–5) were specific as treatment with the full-length parent peptide had no effect on migration or downstream signaling in GN11 cells (7). In our current experiments, we sought to utilize a migration assay that better reflects the external environment that migrating GnRH neurons are exposed to during development. Using a transwell assay coated with matrigel matrix, we demonstrate that GnRH-(1–5) indeed inhibits migration and invasion. Furthermore, targeting GPR173 expression reversed the GnRH-(1–5) effect. Subsequently, we wanted to identify the mechanism of GnRH-(1–5) to inhibit invasion through the matrigel-coated inserts. We focused our analysis on chemokines and modulators of ECM remodeling to identify GnRH-(1–5) targets also implicated in the migratory response. GnRH-(1–5) had no effect on chemokine secretion or MMP-2 and MMP-9 activity. However, we found that GnRH-(1–5) decreased TGF-β secretion to subsequently decrease the amount of bioactive TGF-β present that would normally induce a pro-promote migratory response in GN11 cells. Our findings demonstrate that GnRH-(1–5) can regulate the responsivity of GN11 cells to secreted pro-migratory effectors in the presence of an ECM mimetic. This type of regulation may involve GnRH-(1–5) inhibiting TGF-β secretion in addition to proteinases that process latent TGF-β to its active form.

It is well established that there is complex interplay between the extracellular environment and migrating neurons during development. Secreted factors and ECM remodelers are essential in guiding neurons to their final destination within the CNS. This is evident in the development of the GnRH neuroendocrine system. GnRH neurons are born in the nasal region of the developing brain and require multiple guidance cues for their proper establishment within the basal forebrain (10, 34, 35). Defective GnRH neuronal migration can lead to hypogonadotropic hypogonadism, specifically Kallmann syndrome, where there is a delay in pubertal onset and poor reproductive function (36). The earliest gene to be implicated in defective GnRH neuronal migration was the ECM glycoprotein, anosmin-1 (37–39). It is now known that anosmin-1, in the extracellular environment, can interact with membrane bound heparan sulfate proteoglycans to modulate fibroblast growth factor receptor (FGFR) 1 signaling in migrating GnRH neurons (40–42). In addition, anosmin-1 can form a complex with heparin sulfates and serine proteases to alter the extracellular milieu by regulating the proteolytic activation of ECM growth factors, which in turn can regulate cell proliferation and migration (43). Likewise, in our studies we demonstrate that GnRH-(1–5) inhibits the migration of GN11 cells in the presence of an extracellular environment, suggesting that GnRH-(1–5)-mediated activation of GPR173 may regulate the interaction of ECM proteins and membrane-associated factors to modulate the migratory machinery. The ECM mimetic (matrigel) consists of a proteinaceous mixture of collagen, laminin, fibronectin, and entactin. It is likely that the downstream mechanism of GnRH-(1–5) binding GPR173 requires the availability of these ECM proteins to potentially coordinate and cluster signaling complexes necessary to inhibit migration.

Chemokines are secreted factors that are involved in normal immune function (44) in addition to being implicated in the development of certain cancers (45, 46). With regard to the development of the GnRH neuroendocrine system, certain chemokines have been shown to regulate the migration of GnRH neurons *in vivo* (14, 15, 47, 48) and in *in vitro* studies (30, 49). Therefore, in our current model, we sought to determine whether GnRH-(1–5) regulates the secretion of chemokines, which may act in autocrine mechanisms to regulate the migratory response of GN11 cells. We found that of the chemokines measured (CXCL1, CXCL2, CXCL9, CXCL10, CCL3, CCL4, CCL5, and MCSF), none of their secretion levels were regulated by GnRH-(1–5) treatment. Furthermore, GN11 cells did not express CCL2 and CCL4 while high levels of CXCL1 were detected. These results demonstrate the mechanism of GnRH-(1–5) does not involve this family of chemokines; however, it will be interesting to identify their mechanism in the regulation of GnRH neuronal migration independent of GnRH-(1–5). A comprehensive study measuring the expression of chemokines and their receptors in GnRH neurons, to our knowledge, has not been examined.

The lack of an effect on chemokine release in response to GnRH-(1–5) led us to identify other factors which may play a role in the actions of GnRH-(1–5). Growth factors present in the extracellular environment are well known to regulate the development of the CNS (31, 50). An important growth factor, TGF-β, has been implicated in regulating multiple developmental processes including cell fate and neuronal migration (31, 51, 52). In the adult female rat, the TGF-βRI is expressed in a subset of GnRH neurons (53), and TGF-β has been shown to regulate GnRH expression (54), demonstrating that indeed TGF-β signaling is also important in GnRH biology. However, during development of the GnRH neuroendocrine system, very little is known regarding the effects of TGF-β on the ontogeny and migration of GnRH neurons. In the current study, we found that GN11 cells secrete TGF-β1, 2, and 3; yet, TGF-β1 levels were significantly higher than the other two isoforms. Furthermore, GN11 cells express both TGF-β receptors, TGF-βRI and TGF-βRII. TGF-β is secreted as a latent complex associating with a latency-associated protein and a latent-TGF-β-binding protein, which as a complex is unable to bind its receptor (55). Thus, in the extracellular environment, latent TGF-β can associate with other proteins or be proteolytically processed to generate the bioactive TGF-β for receptor activation (55). Initially, we measured the release of total (latent and active) levels of all three isoforms in GN11 cells in response to GnRH-(1–5). We found that GnRH-(1–5) inhibited total TGF-β1 release but not TGF-β2 or 3. This effect was modest; therefore, we determined whether this decrease in total TGF-β1 release was biologically relevant using an established luciferase assay to measure bioactive TGF-β. In this assay, we treated an MLEC line that stably expresses the TGF-β-responsive plasminogen activator-1 luciferase reporter (21, 22) with conditioned media isolated from GN11 cells treated with or without GnRH-(1–5). Interestingly, GnRH-(1–5) decreased the activation of the reporter construct, demonstrating that GnRH-(1–5) not only decreased total TGF-β levels but also bioactive TGF-β. In the ECM, latent TGF-β can reach high levels and only a small fraction is converted to active TGF-β, which can lead to profound biological effects (55, 56). Our findings suggest that TGF-β may exert a pro-migratory autocrine effect in which GnRH-(1–5) plays a modulatory role as an inhibitor of this pathway.

All TGF-β isoforms are embryonically expressed and thought to play a role in neuronal survival and migration (57). In our model, we demonstrate that GnRH-(1–5) selectively inhibits GN11 cellular migration only in the presence of an ECM mimetic in a GPR173-dependent manner. Furthermore, the downstream mechanism involves decreasing the total and active TGF-β1 levels, leading to a decrease in the pro-migratory effects of TGF-β. The processing of latent TGF-β to its active form is a tightly regulated mechanism that can involve the presence of ECM scaffolding proteins and remodelers. Scaffolding proteins such as collagen, laminin, fibronectin, and entactin can play an integral part in mediating the communication between external cues and GnRH-migrating neurons. For example, in the extracellular environment anosmin-1 can complex with heparin sulfate proteoglycans, proteases, and FGFR1 to modulate intracellular signaling events initiated at the plasma membrane level (40, 42, 43) to in part regulate migration rate. Similarly, our findings suggest that GnRH-(1–5)-activating GPR173 leads not only to a decrease in total TGF-β1 release but also a decrease in the conversion of bioactive TGF-β. It is likely that the mechanism of GnRH-(1–5) requires the presence of scaffolding proteins present in the extracellular environment to cluster or orchestrate a signaling complex that acts to inhibit the processing of latent TGF-β. To identify players in this signaling cascade, we initially measured the activity of the ECM proteases, MMP-2 and MMP-9, which can proteolytically activate TGF-β (32, 33). We found no significant changes in MMP-2 or MMP-9 activity in response to GnRH-(1–5). Therefore, we implemented a high-throughput focused array to measure the expression of a panel of ECM remodelers in GN11 cells treated with GnRH-(1–5). Interestingly, we found a consistent increase in TIMP1 levels, a gene also expressed embryonically in the nasal region, and this increase was confirmed by ddPCR in GN11 cells treated with GnRH-(1–5). TIMP1 belongs to a family of ECM proteins (TIMP1-3) that have many functions in development but primarily act to inhibit proteinase activity (58). Our results indicate that the increase in TIMP1 levels may contribute to the inhibition of latent TGF-β activation by inhibiting the processing enzyme. However, the identity of the processing enzyme(s) is still not clear, but our focused array panel suggests that GN11 cells express many ECM remodelers and proteases, which may be involved. Therefore, it will be important to measure the activity of these remodelers since our focused array is dependent on gene expression levels only. Our future work will focus on continuing to expand on our model that implicates GnRH-(1–5) as a mediator in the complex interplay between the extracellular environment and migrating GnRH neurons.

The complex communication between GnRH neurons and the extracellular environment that enables their proper migration from the nasal region to the CNS remains largely unknown (8, 35). Interestingly, certain GPCRs have been implicated in the pathogenesis of KS and hypogonadism underscoring their significance in the establishment of the mature GnRH neuronal system (13). We have previously demonstrated that GPR173 is expressed in the mouse nasal compartment during development as GnRH neurons are migrating to the CNS (7). Although GPR173 expression was detected in the nasal region, it is still unclear whether individual migrating GnRH neurons express GPR173. In our *in vitro* studies, GPR173 is expressed in GN11 cells, an immortalized GnRH secreting cell line, suggesting that GnRH neurons may also express this receptor; however, this remains to be determined *in vivo*. Nevertheless, our studies suggest that GPR173 activation may play a modulatory role in the migration of GnRH neurons during development *via* the regulation of TGF-β signaling. We propose that the conversion of GnRH to GnRH-(1–5) early in development along the GnRH neuronal migratory path may act in autocrine mechanism to activate GPR173 and subsequently regulate the migration of rate of GnRH-secreting neurons prior to entering the CNS *via* the cribriform plate. Specifically, GnRH-(1–5)-mediated activation of GPR173 may contribute to the brief delay observed in the migration rate of GnRH neurons as they transit into the CNS in addition to GnRH neuronal maturation (35). However, it is also plausible that a physical barrier, change in the extracellular milieu, or other unknown ligand may account for this delay independent of GnRH-(1–5). It has been previously demonstrated that both targeted disruption of either GnRH-1 or the GnRH receptor does not alter the proper distribution of GnRH neurons in the brain (59). These findings indicate that redundant or compensatory mechanisms may govern the modulatory role played by GPR173 in the regulation of GnRH neuronal migration. Interestingly, the recently discovered peptide, phoenixin, has been implicated in the activation of GPR173 to regulate reproductive-related processes (60, 61). It is possible that phoenixin or phoenixin-related peptides if expressed during development may also contribute to the GnRH system developmentally. Whether GnRH-(1–5) or phoenixin indeed is involved in the migration of GnRH neurons remains to be investigated in animal models.

In summary, our current study demonstrates that GnRH-(1–5) binding GPR173 inhibits the migratory and invasive properties of immortalized GnRH neurons. These effects are specific to GnRH-(1–5) as treatment with the full-length GnRH peptide has no effect on GN11 cellular migration (7). In addition, we have demonstrated in other cell lines responsive to GnRH-(1–5) where the GnRH receptor is also expressed that exposure to GnRH analogs had no effect on GnRH-(1–5) action (62). In this study, the ability of GnRH-(1–5) to inhibit the invasion of GN11 cells was dependent on the presence of an ECM mimetic, indicating that the downstream mechanism of GnRH-(1–5) requires the availability of ECM scaffolding proteins such as collagen, laminin, fibronectin, and entactin. We also demonstrate that GnRH-(1–5) inhibits the invasion of GN11 cells by decreasing the pro-migratory effects of TGF-β1 by potentially mobilizing ECM remodelers such as TIMP1. The presence of extracellular scaffolding proteins likely allows for the proper clustering and formation of signaling complexes initiated by GnRH-(1–5) that include latent TGF-β and ECM effectors. Further work is required to identify all players involved in this complex signaling cascade.

## Author Contributions

DL, SM, and TW designed and conducted experiments in addition to writing manuscript. BB and MC-C conducted experiments.

## Disclaimer

The opinions or assertions contained herein are the private ones of the authors and are not to be construed as official or reflecting the views of the Department of Defense or the Uniformed Services University of the Health Sciences.

## Conflict of Interest Statement

The authors declare that the research was conducted in the absence of any commercial or financial relationships that could be construed as a potential conflict of interest.
